# The Association Between FT4/FT3 Ratio and Prognosis in Ischemic Stroke: A Retrospective Cohort Study

**DOI:** 10.1002/brb3.71128

**Published:** 2025-12-10

**Authors:** Guiling Wan, Linhong Mo

**Affiliations:** ^1^ Neurological Rehabilitation Center, Beijing Rehabilitation Hospital Capital Medical University Beijing China

**Keywords:** Ischemic stroke, FT4/FT3 ratio, prognosis, thyroid hormones

## Abstract

**Background and Aim:**

The FT4/FT3 ratio reflects thyroid hormone metabolism and has emerged as a prognostic marker in cardiovascular diseases. However, its role in ischemic stroke (IS) remains unclear. This study aimed to investigate the association between the FT4/FT3 ratio and 3‐month functional outcomes in IS patients.

**Methods:**

We conducted a retrospective cohort study of 199 first‐episode IS patients admitted within 14 days of onset between June 2021 and June 2023. Serum thyroid stimulating hormone (TSH), free triiodothyronine (FT3), and free thyroxine (FT4) were evaluated upon admission. Neurological severity was assessed using the National Institutes of Health Stroke Scale (NIHSS) at admission. Functional outcomes were evaluated using the modified Rankin Scale (mRS) at 3 months post‐stroke. Poor outcome was defined as an mRS score of 3–5. Separate analyses were conducted according to FT4/FT3 ratio and outcome.

**Results:**

Patients were stratified by median FT4/FT3 ratio (3.75) into low (≤ 3.75, *n* = 100) and high (> 3.75, *n* = 99) ratio groups. The high‐ratio group had lower FT3 (4.14 ± 0.52 vs. 4.68 ± 0.52 pg/mL, *p* < 0.001), higher FT4 (17.83 ± 2.10 vs. 15.36 ± 1.69 pmol/L, *p* < 0.001), more diabetes (52.5% vs. 34%, *p* = 0.010), and higher proportion of poor outcomes (46.5% vs. 28%, *p* = 0.007). Receiver operating characteristic (ROC) analysis revealed that the FT4/FT3 ratio demonstrated the highest predictive ability (area under the curve [AUC] = 0.662) with an optimal cut‐off of 3.845. After adjusting for NIHSS scores, age, sex, and vascular risks, the FT4/FT3 ratio remained an independent predictor of poor outcomes (odds ratio [OR] = 2.589, 95% confidence interval [CI]: 1.171 − 5.727, *p* = 0.019). FT4 was a risk factor (OR = 1.324, 95% CI: 1.045 − 1.678, *p* = 0.020), while FT3 showed a nonsignificant protective trend (OR = 0.551, 95% CI: 0.218 − 1.390, *p* = 0.207).

**Conclusion:**

An elevated FT4/FT3 ratio may serve as a novel biomarker for predicting poor outcomes in ischemic stroke, reflecting thyroid hormone metabolic dysfunction that potentially exacerbates inflammation and impairs neuronal repair.

**Limitations:**

This study is limited by its small sample size, single‐center design, and absence of serial hormone measurements.

## Graphical abstract

This study identifies the FT4/FT3 ratio as a novel prognostic biomarker in ischemic stroke. Patients with elevated ratios demonstrated significantly poorer 3‐month functional outcomes. The ratio remained an independent predictor after adjusting for stroke severity, age, and vascular risk factors, reflecting underlying thyroid metabolic dysfunction that impairs recovery.

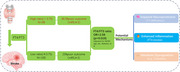

## 1 Introduction

Ischemic stroke (IS) is a leading cause of mortality and long‐term disability worldwide. Despite advances in endovascular recanalization therapies, a significant proportion of stroke survivors experience poor functional outcomes. Recent studies have highlighted the potential role of thyroid dysfunction in stroke pathophysiology and recovery.

The thyroid hormones, thyroxine (T4) and triiodothyronine (T3), play crucial roles in cerebral metabolism and neuroprotection (Rao et al. 2025). The conversion of T4 to the more biologically active T3, mediated by deiodinase enzymes, is a critical regulatory step in thyroid hormone metabolism (Bassols et al. 2011; Maia et al. 2011). While most circulating thyroid hormones are protein‐bound, the free fractions (free thyroxine [FT4] and free triiodothyronine [FT3]) represent the biologically active forms that can cross the blood‐brain barrier.

Emerging evidence suggests that the FT4/FT3 ratio, reflecting the balance between thyroid hormone production and peripheral conversion, may serve as a sensitive indicator of thyroid metabolic status. In cardiovascular diseases, this ratio has demonstrated superior prognostic value compared to isolated hormone measurements (Yuan et al. 2021; Lang et al. 2022; Zhou et al. 2024; Han et al. 2025). However, its relationship with stroke outcomes remains unexplored (Zhang et al. 2024).

This study aimed to investigate whether the FT4/FT3 ratio at admission is associated with 3‐month functional outcomes in patients with first‐episode acute IS, independent of traditional prognostic factors.

## 2 Materials and Methods

### 2.1 Study Design and Participants

**FIGURE 1:**
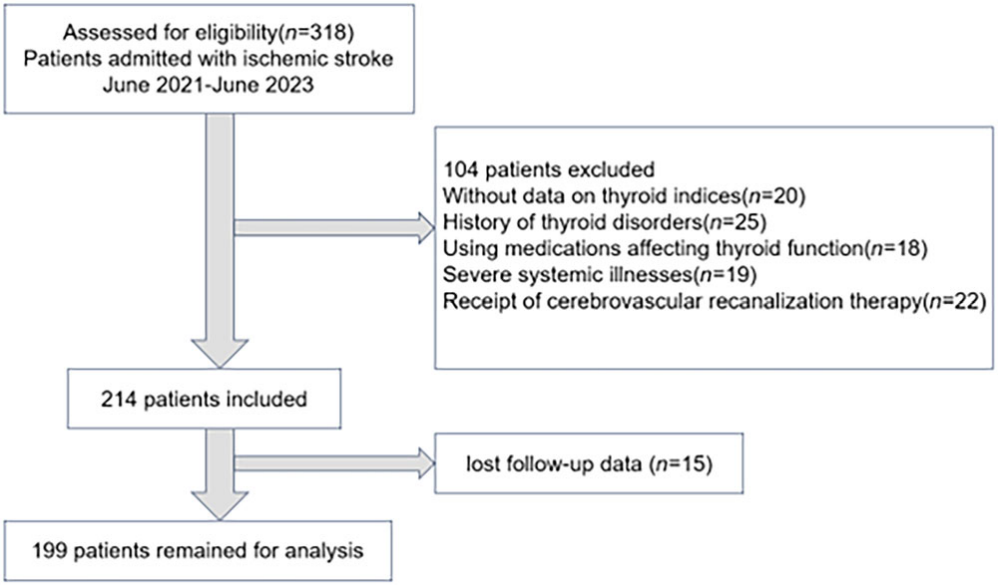
Study flow diagram.

We conducted a retrospective cohort study of consecutive patients admitted to the Neurological Rehabilitation Center at Beijing Rehabilitation Hospital between June 2021 and June 2023. Inclusion criteria are as follows: (1) first‐episode acute IS confirmed by brain magnetic resonance imaging (MRI); (2) admission within 14 days of symptom onset; and (3) availability of complete thyroid function tests at admission. Exclusion criteria are as follows: (1) history of thyroid disorders or pituitary diseases; (2) use of medications affecting thyroid function; (3) severe systemic illnesses (e.g., heart failure, renal failure); (4) incomplete follow‐up data; and (5) previous receipt of cerebrovascular recanalization therapy. The flowchart for the study is shown in Figure 1.

### 2.2 Data Collection

**FIGURE 2:**
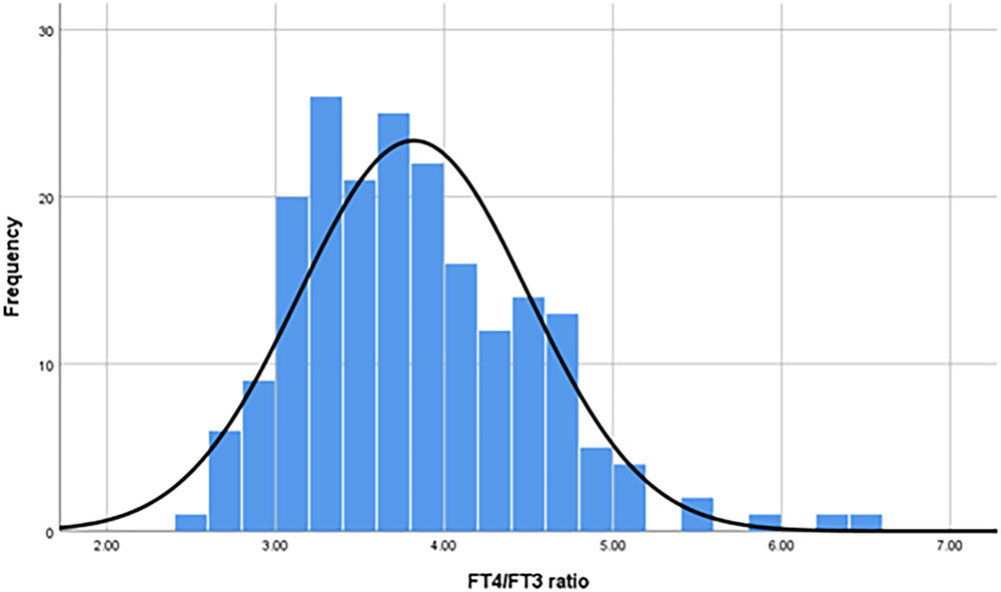
Distribution of FT4/FT3 ratio in ischemic stroke patients.

Baseline demographic characteristics, vascular risk factors, and clinical data were extracted from electronic medical records. All patients underwent standardized laboratory testing within 24 h of admission, including complete blood count, lipid profile, and thyroid function tests (thyroid‐stimulating hormone [TSH], FT3, FT4). The FT4/FT3 ratio was calculated for each patient. Due to the non‐normal distribution of the FT4/FT3 ratio (Figure 2 for histogram), patients were divided into low‐ratio and high‐ratio groups based on the median value (3.75).

Stroke severity was assessed using the National Institutes of Health Stroke Scale (NIHSS) at admission. Functional outcome was evaluated using the modified Rankin Scale (mRS) at 3 months post‐stroke, obtained via outpatient visits or telephone interviews conducted by trained neurologists blinded to thyroid function results. Poor outcome was defined as mRS ≥ 3.

### 2.3 Statistical Analysis

Continuous variables were presented as mean ± standard deviation or median (interquartile range) based on distribution normality. Categorical variables were expressed as frequencies (percentages). Between‐group comparisons were performed using Student's *t*‐test, the Mann–Whitney U test, or chi‐square test as appropriate.

Univariate and multivariate logistic regression analyses were conducted to identify factors associated with poor outcome. Variables with *p* < 0.1 in univariate analysis or clinically relevant factors were included in the multivariate model. Results were expressed as odds ratios (ORs) with 95% confidence intervals (CIs).

Receiver operating characteristic (ROC) curve analysis was performed to evaluate the discriminative ability of thyroid function parameters for poor outcomes. Area under the curve (AUC) values were calculated with 95% CIs. Optimal cut‐off points were determined using Youden's index.

All analyses were performed using SPSS 26.0, with statistical significance set at *p* < 0.05 (two‐tailed).

## 3 Result

### 3.1 Baseline Characteristics

The study included 199 patients (mean age 58.9 ± 11.3 years, 84.9% male). Using the median FT4/FT3 ratio (3.75) as the cutoff, patients were divided into low‐ratio (≤ 3.75, *n* = 100) and high‐ratio (> 3.75, *n* = 99) groups (Table 1).

**TABLE 1:** Baseline characteristics of the study population grouped by FT4/FT3 ratio.

	FT4/FT3 (≤ 3.75)*n* = 100	FT4/FT3 (> 3.75)*n* = 99	*p*‐value
Male, *n* (%)	92 (92%)	77 (77.8%)	0.006^＊^
Age, years	58.94 ± 10.01	58.85 ± 12.61	0.955
BMI, kg/m^2^	26.11 ± 3.27	25.60 ± 3.62	0.290
**Medical history**			
Hypertension, *n* (%)	70 (70%)	79 (79.8%)	0.141
Diabetes, *n* (%)	34 (34%)	52 (52.5%)	0.010^＊^
Dyslipidemia, *n* (%)	61 (61%)	62 (62.6%)	0.884
Coronary heart disease, *n* (%)	19 (19%)	14 (14.1%)	0.446
Family history of cardiocerebrovascular disease, *n* (%)	38 (38%)	35 (35.4%)	0.769
Smoking, *n* (%)	66 (66%)	57 (57.6%)	0.245
Alcohol use, *n* (%)	48 (48%)	35 (35.4%)	0.085
**Laboratory results**			
WBC, ×10⁹/L	6.72 (5.56, 7.93)	6.83 (5.90, 8.28)	0.145
Hemoglobin, g/L	150 (141, 156)	142 (132, 153)	0.004^＊^
Triglycerides, mmol/L	1.60 (1.12, 2.09)	1.53 (1.12, 2.33)	0.680
Cholesterol, mmol/L	3.71 (3.24, 4.34)	4.04 (3.43, 4.63)	0.092
HDL‐C, mmol/L	0.94 (0.83, 1.11)	0.96 (0.86, 1.09)	0.788
LDL‐C, mmol/L	2.43 ± 0.83	2.71 ± 0.93	0.036^＊^
Homocysteine, umol/L	14.2 (11.8, 16.9)	13.5 (10.60, 17.63)	0.134
**Thyroid function**			
FT3, pmol/L	4.68 ± 0.52	4.14 ± 0.52	˂ 0.001^＊^
FT4, pmol/L	15.36 ± 1.69	17.83 ± 2.10	˂ 0.001^＊^
TSH, mIU/L	1.89 (1.25, 2.77)	1.51 (1.04, 2.35)	0.022^＊^
**Clinical scores**			
Admission NIHSS	3 (1, 4.75)	3 (1, 8)	0.064
3‐month mRS	1 (1, 3)	2 (1, 4)	0.001^＊^

*Note*: Values are presented as mean ± standard deviation, median (interquartile range), or number (%).

Abbreviations: BMI, body mass index; FT3, free triiodothyronine; FT4, free thyroxine; HDL‐C, high‐density lipoprotein cholesterol; LDL‐C, low‐density lipoprotein cholesterol; mRS, modified Rankin Scale; NIHSS: National Institutes of Health Stroke Scale; TSH, thyroid‐stimulating hormone; WBC, white blood cell.

*p < 0.05.

The high‐ratio group had significantly lower FT3 levels, higher FT4 levels, and higher prevalence of diabetes (all *p* < 0.05). There was no statistically significant difference in NIHSS score at admission between the two groups. The 3‐month mRS score was significantly higher in the high‐ratio group than in the low‐ratio group (*p* = 0.001).

### 3.2 Functional Outcomes

**FIGURE 3:**
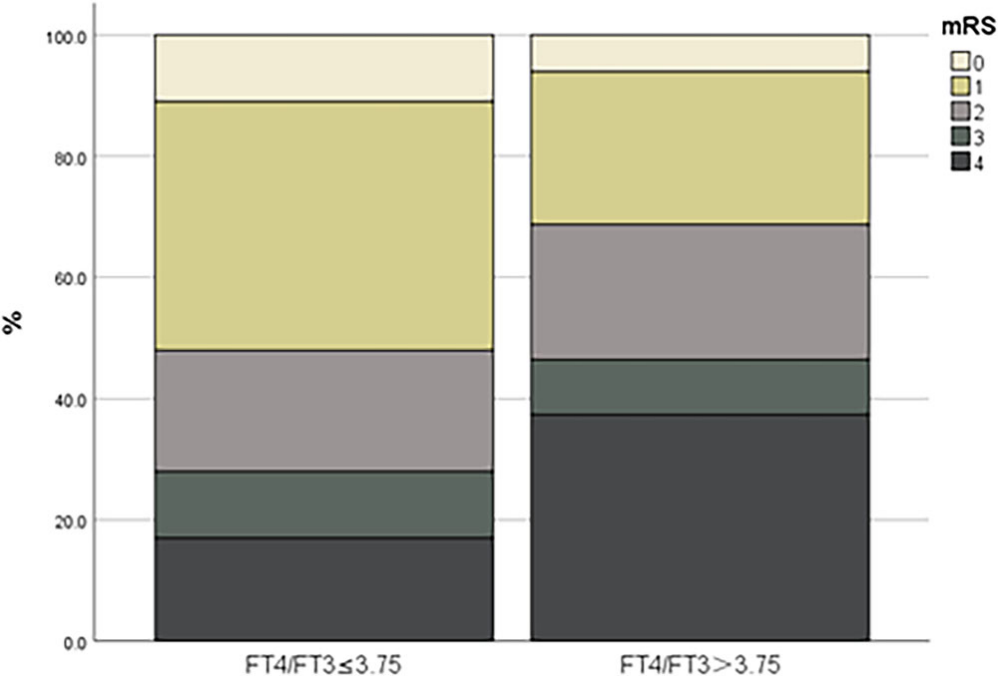
Distribution of 3‐month mRS scores stratified by FT4/FT3 ratio.

At the 3‐month follow‐up, patients in the high‐ratio group had significantly worse functional outcomes (median mRS 2 vs. 1, *p* = 0.001). The proportion of poor outcomes (mRS ≥ 3) was nearly twice as high in the high ratio group (46.5% vs. 28%, *p* = 0.007). The detailed distribution of mRS scores in both groups is presented in Figure 3.

### 3.3 Predictors of Poor Outcomes

#### 3.3.1 Baseline Characteristics Stratified by 3‐Month Functional Outcomes

Table 2 shows statistically significant differences between the good and poor outcome groups in admission NIHSS scores, white blood cell counts, FT4, FT3, and FT4/FT3 ratio (all *p* < 0.05).

**TABLE 2:** Baseline Characteristics according to the functional status at 3 months.

	Good outcome (mRS ˂ 3)*n* = 125	Poor outcome (mRS ≥ 3)*n* = 74	*p*‐value
Age, years	58.33 ± 11.46	59.85 ± 11.25	0.363
Male, *n* (%)	103 (82.4%)	66 (89.2%)	0.224
BMI, kg/m^2^	25.81 ± 3.41	25.94 ± 3.53	0.787
Admission NIHSS	2 (1, 3)	7 (4, 9)	˂ 0.001^＊^
**Medical history**			
Hypertension, *n* (%)	94 (75.2%)	55 (74.3%)	1.000
Diabetes, *n* (%)	49 (39.2%)	37 (50%)	0.142
Dyslipidemia, in (%)	71 (56.8%)	52 (70.3%)	0.070
Coronary heart disease, *n* (%)	21 (16.8%)	12 (16.2%)	1.000
Family history of cardiocerebrovascular disease, *n* (%)	49 (39.2%)	24 (32.4%)	0.364
Smoking, *n* (%)	52 (41.6%)	31 (41.9%)	1.000
Alcohol use, *n* (%)	79 (63.2%)	44 (59.5%)	0.652
**Laboratory results**			
WBC, ×10⁹/L	6.54 (5.42, 7.49)	7.58 (6.30, 8.78)	˂ 0.001^＊^
Hemoglobin, g/L	148 (135, 153)	149 (137, 157)	0.315
Triglycerides, mmol/L	1.61 (1.12, 2.22)	1.51 (1.12, 2.25)	0.953
Cholesterol, mmol/L	3.82 (3.39, 4.53)	3.87 (3.30, 4.42)	0.645
HDL‐C, mmol/L	0.97 (0.86, 1.11)	0.94 (0.83, 1.09)	0.322
LDL‐C, mmol/L	2.55 ± 0.89	2.57 ± 0.88	0.873
Homocysteine, umol/L	13.55 (10.98, 16.93)	14.40 (11.50, 17.65)	0.333
**Thyroid function**			
TSH, mIU/L	1.71 (1.15, 2.59)	1.79 (1.17, 2.56)	0.734
FT4, pmol/L	16.21 ± 2.13	17.23 ± 2.36	0.002^＊^
FT3, pmol/L	4.47 ± 0.58	4.30 ± 0.59	0.043^＊^
FT4/FT3	3.63 (3.26, 4.08)	3.85 (3.56, 4.57)	˂ 0.001^＊^

*Note*: Values are presented as mean ± standard deviation, median (interquartile range), or number (%). Abbreviations as in Table 1.

*p < 0.05.

#### 3.3.2 ROC Curve Analysis for Thyroid Parameters

**FIGURE 4:**
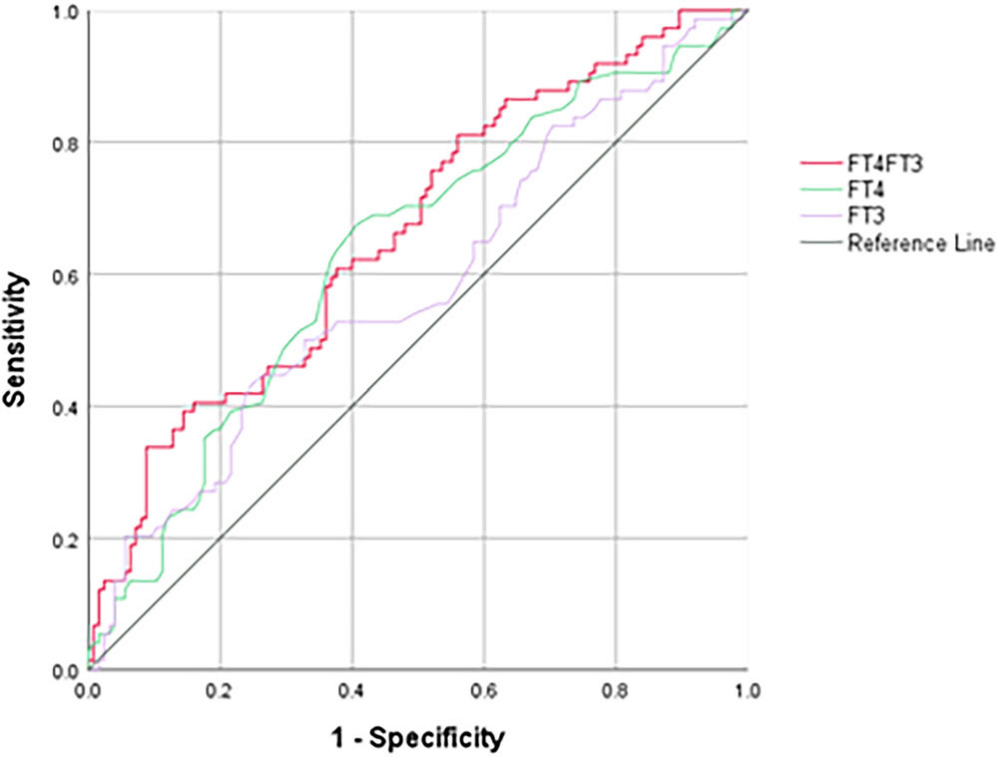
ROC curves of thyroid function parameters for predicting poor 3‐month outcomes.

The predictive performance of thyroid function parameters for poor 3‐month outcomes was evaluated using ROC curve analysis (Figure 4). The FT4/FT3 ratio demonstrated the highest discriminative ability with an AUC of 0.662 (95% CI: 0.585 − 0.739, *p* < 0.001), followed by FT4 (AUC = 0.632, 95% CI: 0.552 − 0.712, *p* = 0.002) and FT3 (AUC = 0.583, 95% CI: 0.500 − 0.666, *p* = 0.051). Optimal cut‐off values determined by Youden's index were 3.845 for the FT4/FT3 ratio (sensitivity 51.4%, specificity 64.0%, Youden's index = 0.154).

#### 3.3.3 Logistic Regression Analysis of Prognostic Factors

Univariate regression analysis identified admission NIHSS score, white blood cell count, and FT4 levels as significant risk factors for poor prognosis (*p* < 0.05), while FT3 demonstrated a protective effect (*p* < 0.05). Variables with *p* < 0.1 in univariate analysis, along with clinically relevant factors (age and sex), were included in the multivariate model. The final analysis revealed that higher admission NIHSS scores (OR 2.419, 95% CI: 1.843 − 3.176, *p* < 0.001) and elevated FT4 levels (OR 1.324, 95% CI: 1.045 − 1.678, *p* = 0.020) were independent risk factors for unfavorable outcomes in patients with IS (Table 3).

**TABLE 3:** Univariate and multivariate logistic regression analysis of factors associated with poor outcome.

	Crude OR(95% CI)	*p*‐value	Adjust OR(95% CI)	*p*‐value
Age	1.012 (0.986, 1.038)	0.361	1.014 (0.969, 1.060)	0.553
Male	0.567 (0.239, 1.349)	0.200	1.316 (0.358, 4.839)	0.680
Dyslipidemia	1.798 (0.975, 3.313)	0.060	2.631 (0.882, 7.853)	0.083
Admission NIHSS	2.228 (1.784, 2.783)	<0.001^＊^	2.419 (1.843, 3.176)	˂ 0.001^＊^
WBC	1.433 (1.186, 1.733)	<0.001^＊^	1.347 (0.997, 1.819)	0.052
FT4	1.228 (1.074, 1.404)	0.003^＊^	1.324 (1.045, 1.678)	0.020^＊^
FT3	0.595 (0.358, 0.989)	0.045^＊^	0.551 (0.218, 1.390)	0.207

Abbreviations: CI, confidence interval; FT3, free triiodothyronine; FT4, free thyroxine; NIHSS, National Institutes of Health Stroke Scale; OR, odds ratio; WBC, white blood cell.

*p < 0.05.

#### 3.3.4 Logistic Regression Models for Poor Prognosis

Table 4 presents that even after adjusting for age, sex, common clinical risk factors (including hypertension, diabetes, and dyslipidemia), and admission NIHSS scores, patients with higher FT4/FT3 ratios maintained a significantly elevated risk of poor prognosis (OR 2.589, 95% CI: 1.171 − 5.727, *p* = 0.019).

**TABLE 4:** Univariate and multivariable logistic regression models for poor prognosis.

Model	OR(95% CI)	*p*‐value
Univariate	2.495 (1.553, 4.008)	˂ 0.001^＊^
Model 1	2.731 (1.664, 4.483)	˂ 0.001^＊^
Model 2	2.764 (1.647, 4.639)	˂ 0.001^＊^
Model 3	2.589 (1.171, 5.727)	0.019^＊^

*Note*: Model 1: adjusted for age and sex; Model 2: additionally adjusted for hypertension, dyslipidemia, diabetes mellitus, coronary artery disease, family history of cardiovascular disease, smoking status; and Model 3: adjusted for variables in Model 2 plus admission NIHSS scores.

*p < 0.05.

## 4 Discussion

This study is the first to systematically investigate the relationship between the FT4/FT3 ratio upon admission and 3‐month functional outcomes in patients with first‐episode IS. The core findings indicate that the FT4/FT3 ratio not only demonstrated superior predictive performance compared to individual thyroid hormones but also remained an independent predictor after adjusting for various prognostic factors, including NIHSS scores.

### 4.1 Theoretical Basis of the FT4/FT3 Ratio in Resolving Contradictions in Previous Studies

Previous studies on the relationship between thyroid hormones and stroke prognosis have shown significant contradictions. On one hand, most studies have indicated that low T3 syndrome is associated with poor prognosis (Bunevicius et al. 2015; Jiang et al. 2017; Lamba et al. 2018), with low T3 levels linked to more severe strokes, complicated hospital courses, and higher mortality (Alevizaki et al. 2007; Ambrosius et al. 2011; Sidorov et al. 2023; Pang et al. 2024; Di Vincenzo et al. 2025). On the other hand, some scholars have proposed that low T3 may be an adaptive energy‐conserving response during illness (Maiden and Torpy 2019), and some have even found that hypothyroidism in stroke is associated with favorable outcomes (Akhoundi et al. 2011).

Similarly, findings regarding T4 are also inconsistent. Most research has not found a significant association between T4 and stroke prognosis (Suda et al. 2018; Murolo et al. 2022). Some studies have found that higher T4 is associated with poor prognosis (Xie et al. 2023), while others have linked low T4 to mortality, cognitive impairment, and post‐stroke depression (Alevizaki et al. 2007). These contradictions may stem from the inability of individual hormone levels to fully reflect the overall state of thyroid metabolism.

In this study, the FT4/FT3 ratio demonstrated better discriminative ability compared to FT3 and FT4, indicating that this ratio can more sensitively capture the essence of thyroid metabolic disturbances (Zhang et al. 2024). A high ratio reflects a state of relative T3 deficiency due to impaired conversion of T4 to T3, and this state may more accurately predict prognosis than changes in individual hormone levels alone (Maia et al. 2011).

### 4.2 Underlying Pathophysiological Mechanisms

A high FT4/FT3 ratio may influence stroke prognosis through multiple mechanisms:

#### 4.2.1 Impaired Neuroprotective Mechanisms

Relative T3 deficiency may weaken its neuroprotective effects (Finck et al. 2023). Basic research has shown that FT3 protects ischemic neurons by regulating mitophagy, promoting glutamate uptake, and expressing brain‐derived neurotrophic factor (Losi et al. 2008; Shulga et al. 2009). Additionally, T3 accelerates glucose transport to brain cells, enhances brain connectivity, and increases cerebrovascular density (Badaut et al. 2002; Shulga et al. 2009). Impairment of these mechanisms may significantly affect neural repair processes.

#### 4.2.2 Exacerbated Inflammatory Response

Relative FT4 excess may exacerbate inflammatory damage by activating pro‐inflammatory pathways such as NF‐κB (Jiang et al. 2017). Elevated T4 may inhibit T3‐converting enzymes (deiodinase D2), further worsening the state of relative T3 deficiency (Jiang et al. 2017). This “double‐hit” mechanism of lacking neuroprotection and activated inflammation explains why the ratio has better predictive value than individual hormones.

#### 4.2.3 Synergistic Effects of Metabolic Disorders

The significantly higher prevalence of diabetes in the high‐ratio group in this study suggests a potential synergistic effect between thyroid hormone metabolic disturbances and glucose metabolism abnormalities. Thyroid hormones influence various metabolic pathways, including insulin sensitivity, fatty acid oxidation, and fat storage (Mancino et al. 2021), which may collectively constitute the metabolic basis for poor outcomes.

### 4.3 Clinical Significance and Application Value

The optimal cut‐off value of 3.845 determined by ROC analysis provides a practical tool for identifying high‐risk patients in clinical practice. It is particularly noteworthy that this indicator remained an independent predictor even after adjusting for inflammatory markers (white blood cell count), supporting its role beyond merely reflecting inflammatory status.

Regarding the phenomenon of poor outcomes in patients with “normal thyroid function,” we propose that traditional normal ranges may fail to identify functional thyroid metabolic disturbances. Even when FT3 and FT4 are within normal ranges, an abnormal ratio can still reflect altered thyroid hormone activity at the tissue level.

To clarify the biological mechanisms underlying this prognostic association, could thyroid hormone replacement therapy improve outcomes in this population? Basic experiments show T3 supplementation benefits stroke animals (Mdzinarishvili et al. 2013; Sayre et al. 2017; Ullrich et al. 2025); however, there are no relevant studies on T3 therapy in stroke patients, which requires further exploration.

## 5 Limitations and Future Directions

The relationship between thyroid hormones and stroke is complex (Neidert et al. 2011; Bunevicius et al. 2014; Dhital et al. 2017), and the severity, stage, and duration of stroke can influence this relationship (Dhital et al. 2017; Keshavarz and Dehghani 2017; Lamba et al. 2018; Maiden and Torpy 2019). Limitations of this study include (1) thyroid function tests were obtained during the acute phase, with no data from hyperacute or recovery phases, precluding evaluation of FT4/FT3 ratio evolution over the disease course and its association with prognosis; (2) the single‐center design and sample size limitations may affect the generalizability of the results; the significant gender imbalance (84.9% male) limits the applicability of the findings to female patients; and (3) while admission NIHSS scores were adjusted for, the lack of data on infarct volume and specific locations means that the confounding effects of these factors cannot be fully excluded. Future multi‐center prospective studies with larger, balanced cohorts should incorporate serial hormone measurements, detailed neuroimaging data, and inflammatory biomarkers to validate these findings and elucidate the underlying mechanisms.

## 6 Conclusion

This study establishes the FT4/FT3 ratio as a novel biomarker for prognosis in IS. By integrating multidimensional information on thyroid hormone metabolism, this indicator provides new insights for resolving contradictory findings in previous studies. Future multicenter prospective studies should validate this finding in more balanced populations and incorporate dynamic hormone monitoring, detailed imaging parameters, and inflammatory markers to further elucidate the potential value of the FT4/FT3 ratio in stroke prognosis assessment and personalized treatment.

## Author Contributions

**Guiling Wan**: conceptualization, data curation, formal analysis, investigation, methodology, project administration, writing – original draft, writing – review and editing. **Linhong Mo**: data curation, formal analysis, methodology, project administration, resources, writing – original draft, writing – review and editing.

## Funding

The authors have nothing to report.

## Ethics Statement

The protocol of this study was approved by Ethics Committee of Beijing Rehabilitation Hospital (Approval No.:2025bkky‐051). All methods were performed in accordance with the Declaration of Helsinki.

## Conflicts of Interest

The authors declare no conflicts of interest.

## Data Availability

The data that support the findings of this study are available from the corresponding author upon reasonable request.
